# Game Over for the Baseline: Influenza Hospitalization Patterns Before, During, and After the COVID-19 Pandemic (FluSurv-NET, 2009–2025)

**DOI:** 10.3390/idr18030061

**Published:** 2026-06-19

**Authors:** Hayden D. Hedman

**Affiliations:** 1 School of Computer Science, Georgia Institute of Technology, Atlanta, GA 30332, USA; hhedman3@gatech.edu; 2 Summit County Local Public Health Agency, Frisco, CO 80443, USA

**Keywords:** influenza surveillance, post-pandemic epidemiology, machine learning, anomaly detection, time-series forecasting, seasonal decomposition, health disparities, FluSurv-NET, population-based surveillance, applied epidemiology

## Abstract

Background/Objectives: The trajectory of influenza hospitalization burden from pre-COVID-19 pandemic baseline through post-pandemic recovery remains poorly characterized at the national level. This study characterized phase-stratified burden and seasonal structure, quantified racial and ethnic disparities, and assessed whether post-pandemic seasons represent anomalous departures from pre-pandemic expectations. Methods: Sixteen complete seasons of FluSurv-NET surveillance data (2009–2010 through 2024–2025; 509 observation weeks) were analyzed across pre-pandemic, disruption, and recovery phases using OLS regression with effect-size estimation, bootstrapped age-adjusted rate ratios, seasonal-trend decomposition (STL), Prophet time-series forecasting, and Isolation Forest anomaly detection. Results: Mean peak weekly hospitalization rate nearly doubled from pre-pandemic to recovery (5.1 to 11.1 per 100,000), cumulative seasonal burden increased from 46.3 to 87.0 per 100,000, and median peak timing advanced from MMWR week 9 to week 50. STL decomposition revealed a marked shift from weak pre-pandemic seasonality (Fs = 0.14) to substantially stronger annual regularity (Fs = 0.98) across three recovery seasons, with threefold amplitude increase. Non-Hispanic Black persons had rate ratios of 1.72, 2.16, and 1.99 relative to White persons across phases; American Indian and Alaska Native persons showed the highest disruption-phase ratio (2.24, 95% CI 1.90–3.53), based on two contributing seasons. A flat-growth Prophet model detected first exceedance in February 2020, outperforming a linear-growth specification on held-out validation. Isolation Forest identified 2017–2018, 2023–2024, and 2024–2025 as robust anomalies across all contamination thresholds. Conclusions: Post-COVID-19 pandemic influenza recovery is characterized by intensified and restructured seasonality, persistent racial and ethnic disparities, and anomalous burden exceeding pre-pandemic projections, identified independently by time-series forecasting and unsupervised anomaly detection.

## 1. Introduction

Seasonal influenza remains a substantial cause of morbidity, hospitalization, and mortality in the United States, with burden concentrated among adults aged 65 years and older, young children, and individuals with underlying medical conditions [1,2]. The Influenza Hospitalization Surveillance Network (FluSurv-NET) [3], a population-based platform covering approximately 10% of the US population across 14 states, has provided the primary infrastructure for tracking laboratory-confirmed influenza hospitalization rates since the 2009–2010 pandemic season [3]. Despite this surveillance infrastructure, the full epidemiological trajectory from the pre-pandemic baseline through pandemic disruption and into the post-pandemic recovery period has not been comprehensively characterized at the national level using the complete FluSurv-NET time series.

The COVID-19 pandemic profoundly disrupted influenza transmission dynamics. Non-pharmaceutical interventions implemented beginning in 2020 suppressed influenza circulation to near-zero levels during the 2020–2021 season, with an extended period of minimal transmission giving rise to what has been termed immunological debt: a population-level reduction in naturally acquired immunity due to the absence of antigenic exposure over multiple seasons, resulting in an expanded susceptible pool when transmission resumed [4,5,6]. Evidence for this phenomenon has been documented across multiple respiratory pathogens in the post-COVID-19 pandemic period [5,6,7], though data specifically characterizing the magnitude, trajectory, and structural character of influenza’s post-pandemic recovery in the United States remain limited [8,9]. The 2022–2023 through 2024–2025 seasons represent the first complete recovery-phase influenza seasons available for systematic longitudinal analysis.

Racial and ethnic disparities in influenza hospitalization burden have been documented persistently across seasons, with non-Hispanic Black and American Indian and Alaska Native populations consistently experiencing higher hospitalization rates than non-Hispanic White populations [10,11]. Whether the disruption and recovery periods widened or attenuated pre-existing disparities remains an open empirical question.

Existing analyses of post-pandemic influenza dynamics have largely focused on single seasons, specific subpopulations, or burden estimation methodologies rather than longitudinal characterization of the full pre-pandemic through recovery trajectory [7,9]. Longitudinal characterization of influenza hospitalization burden and seasonal structure across the full pandemic transition remains incomplete, and multi-method analytic approaches applied to population-based surveillance data spanning this period are limited.

To address this gap, longitudinal analysis of FluSurv-NET surveillance data was applied to examine: (1) phase-stratified influenza hospitalization burden and seasonal structure across the pre-pandemic, disruption, and recovery periods; (2) racial and ethnic disparities in hospitalization rates and their evolution across phases; and (3) whether recovery-phase seasons represent anomalous departures from pre-pandemic expectations. Post-pandemic influenza recovery was characterized by compressed but intensified seasonality, persistent and widened racial and ethnic disparities, and anomalous peak burden in the two most recent complete seasons exceeding pre-pandemic projections across multiple independent analytic methods.

## 2. Materials and Methods

### 2.1. Study Design and Data Source

Population-based longitudinal surveillance data were obtained from the Influenza Hospitalization Surveillance Network (FluSurv-NET) (Supplemental Materials S1), operated by the Centers for Disease Control and Prevention (CDC) as a component of the Respiratory Virus Hospitalization Surveillance Network (RESP-NET) [3]. FluSurv-NET conducts active, laboratory-confirmed influenza hospitalization surveillance across more than 90 counties in 14 states, representing approximately 34 million persons and an estimated 10% of the United States of America population across nine Department of Health and Human Services regions [3].

A case was defined as a resident of a FluSurv-NET catchment area who received a positive influenza laboratory test ordered by a healthcare professional within 14 days before or during hospitalization. Acceptable laboratory methods included viral culture, direct or indirect fluorescent antibody staining, rapid antigen testing, and molecular assay [3,12]. No additional inclusion or exclusion criteria were applied beyond CDC’s published case definition and suppression rules. Data were extracted from FluView Interactive on 7 March 2026 [3]. All analyses were restricted to the Entire Network catchment stratum to ensure geographic comparability across seasons; sub-network and state-level strata were excluded to avoid differential catchment effects introduced by changes in network composition over time.

### 2.2. Study Period and Phase Classification

The analytic dataset spanned the 2009–2010 through 2024–2025 influenza seasons, comprising 16 complete seasons of population-based surveillance (Supplemental Materials S1 and S2). The in-progress 2025–2026 season was retained for descriptive summaries but excluded from all time-series models and regression analyses requiring complete seasonal data.

Seasons were classified into three a priori epidemiological phases. The pre-pandemic baseline phase comprised the 2009–2010 through 2018–2019 seasons (ten seasons), representing the normative range of influenza hospitalization burden prior to SARS-CoV-2 emergence. The pandemic disruption phase comprised the 2019–2020 through 2021–2022 seasons (three seasons), characterized by suppressed influenza circulation attributable to non-pharmaceutical interventions, behavioral change, and competitive viral interference during the COVID-19 pandemic [13,14,15]. The post-pandemic recovery phase comprised the 2022–2023 season onward (three complete seasons: seasons: 2022–2023, 2023–2024, and 2024–2025), reflecting re-establishment of seasonal influenza dynamics following relaxation of pandemic-era measures.

The 2009–2010 influenza A(H1N1) pandemic season was retained in all primary analyses but flagged as a binary covariate in sensitivity models to account for its atypical epidemiological profile [12]. Weekly hospitalization rates for the 2020–2021 season were suppressed by CDC due to low counts and data quality concerns arising from COVID-19 co-circulation; this season contributed 31 observation weeks with non-null cumulative rates but null weekly rates. These observations were retained as confirmed structural absences and were not imputed; rate-based analyses for 2020–2021 relied exclusively on cumulative rates.

### 2.3. Surveillance Sampling and Denominator Considerations

Two methodological discontinuities require consideration for valid inference across the full study period. Beginning with the 2017–2018 season, FluSurv-NET implemented probability-based sampling for clinical data collection: patients aged 50 and older were age-stratified and sampled below 100%, while all in-hospital deaths were captured in full regardless of age [3]. Clinical data elements subject to this design should be interpreted as weighted estimates rather than complete case counts.

A second discontinuity affects population denominators used to compute hospitalization rates: seasons prior to 2020–2021 used National Center for Health Statistics bridged-race population estimates, while seasons from 2020 to 2021 onward used unbridged U.S. Census Bureau estimates following discontinuation of bridged-race products [3]. This transition introduces a comparability limitation for absolute rate comparisons across the disruption boundary; within-phase comparisons are internally consistent. Cross-phase absolute rate comparisons were examined with a denominator-version indicator variable included in sensitivity models.

### 2.4. Data Management and Variable Construction

Raw surveillance data were preprocessed to address suppression artifacts, temporal indexing, and season-boundary ambiguities inherent to multi-season public health surveillance extracts. CDC suppression markers were replaced with missing values; confidence interval fields were absent across all strata and excluded from analysis. Boundary-week duplicates arising at the MMWR week 53/week 1 transition within a single season (two such observations across the entire dataset) were de-duplicated by retaining the higher weekly rate to preserve peak burden estimates [3]. The 2023–2024 season comprises 52 observation weeks rather than the standard approximately 30, reflecting an optional FluSurv-NET year-round site-extension protocol implemented for that season; this design feature is acknowledged in interpretation and addressed via a sensitivity analysis truncating the 2023–2024 record to its standard October–April surveillance window. A continuous weekly time series was constructed using MMWR epiweeks. The 2020–2021 season was confirmed to reflect genuine near-zero influenza activity rather than data loss, based on non-null cumulative rate records [3].

All time-series and phase-level inference used the overall network stratum. Stratified subsets by age group, sex, race/ethnicity, and virus type were used for descriptive and disparity analyses. All analyses were conducted in Python version 3.11 (Python Software Foundation, Wilmington, DE, USA) using pandas, NumPy, statsmodels, scikit-learn, and Prophet. A fixed random seed was applied to all stochastic procedures to ensure reproducibility (Supplemental Materials S1).

### 2.5. Descriptive Analysis

Descriptive summaries were computed across five stratification dimensions: overall network, five standard CDC age groups (0–4, 5–17, 18–49, 50–64, and ≥65 years), sex (male and female), race/ethnicity (White, Black, Hispanic/Latino, Asian/Pacific Islander, and American Indian/Alaska Native), and influenza virus type (Influenza A, Influenza B, A(H1N1)pdm09, and A(H3N2)) [3].

The variance-to-mean ratio of weekly hospitalization rates was computed within each stratum prior to summarization to evaluate distributional properties and inform the choice of central tendency measure [16]. Median with interquartile range was used as the primary phase-level summary, with mean and standard deviation as secondary estimates. Season-level metrics were computed first for each stratum and phase, including peak weekly rate, peak MMWR week, end-of-season cumulative rate, and active season duration, defined as the number of weeks with a weekly rate ≥ 0.1 per 100,000 population. These season-level values were then averaged within phase to prevent seasons with longer observation windows from exerting disproportionate influence on phase estimates. The 2020–2021 season was excluded from weekly rate summaries but retained in cumulative rate summaries.

### 2.6. Race/Ethnicity Disparity Analysis

Age-adjusted cumulative hospitalization rate ratios were estimated by race/ethnicity and epidemiological phase using the White population as the reference group. The outcome for each season was the end-of-season maximum age-adjusted cumulative hospitalization rate. Phase-level rate ratios were calculated as the ratio of phase means across seasons, with 95% confidence intervals derived by nonparametric bootstrap resampling of season-level pairs (1000 iterations, percentile method). The 2020–2021 season was excluded because all race-stratified weekly rates were structurally suppressed. Cross-phase comparisons of absolute rate levels were interpreted with caution, given the denominator discontinuity described in Section 2.3; within-phase rate ratios are the primary inferential targets.

### 2.7. Phase-Level Regression Analysis

The overall-stratum weekly rate series was aggregated to the season level, yielding one mean weekly rate per season, and regressed on phase dummy variables using ordinary least squares with the pre-pandemic phase as the reference category (Supplementary Materials S3). The analytic sample comprised 15 seasons after excluding 2020–2021 and the partial 2025–2026 season. A sensitivity model additionally adjusted for denominator version and the 2009–2010 pandemic indicator. Effect sizes were quantified as Cohen’s d computed pairwise between phases using pooled standard deviation [17]. The gap between unadjusted and adjusted R^2^ in the primary model reflects limited degrees of freedom at *n* = 15 seasons; effect size estimation is foregrounded accordingly.

### 2.8. Linear Mixed-Effects Model

A linear mixed-effects model was fit to weekly hospitalization rates with within-season week number as a fixed effect and a season-level random intercept, estimated by restricted maximum likelihood. Phase was not included as a fixed effect, as phase classification is invariant within season and would be fully absorbed by the season-level random intercept. The within-season week number coefficient is interpreted as the average within-season rate trajectory across all modeled seasons, independent of between-season burden differences captured by the random intercept.

### 2.9. Seasonal-Trend Decomposition

Seasonal-trend decomposition using LOESS (STL) was applied separately to the pre-pandemic series (2009–2010 through 2018–2019; 300 weeks) and the post-pandemic recovery series (2022–2023 through 2024–2025; 112 weeks) to isolate structural changes in seasonality independent of trend [18]. The pandemic disruption phase was excluded from both fits. Decomposition used a period of 52 weeks with robust LOESS fitting. Seasonal amplitude was quantified as the interquartile range of the seasonal component. Strength of seasonality (Fs) and strength of trend (Ft) were computed [19]. Post-pandemic series estimates should be interpreted with the constraint of three contributing seasons; the consistency of directional shifts across all four decomposition metrics supports the observed structural change.

### 2.10. Time-Series Forecasting and Anomaly Detection

A Prophet time-series model was fit to the overall-stratum weekly rate series using all non-null pre-pandemic weeks as training data (300 weeks; 27 September 2009 to 21 April 2019) to generate out-of-sample forecasts representing expected rates under pre-pandemic seasonal dynamics [20,21]. The primary model specified flat growth with additive yearly seasonality, a changepoint prior scale of 0.05, 15 potential changepoints, and 95% prediction intervals derived from 1000 uncertainty samples. A flat growth specification was selected over linear growth to avoid extrapolating the pre-pandemic upward trend into the forecast period, which would inflate projected baselines and delay anomaly detection. A sensitivity model substituting linear growth was fit to the same training data. Both models were evaluated on a held-out validation season (2018–2019) withheld prior to out-of-sample projection (Supplementary Materials S4). Detection stability was assessed via leave-one-out refitting, excluding each pre-pandemic season in turn and recording the first exceedance date (see Supplementary Materials S5). The first post-training week in which the observed rate exceeded the upper bound of the 95% prediction interval was identified as the anomaly onset date for each model.

### 2.11. Isolation Forest Anomaly Detection

The Isolation Forest algorithm was applied to a season-level feature matrix constructed from the overall-stratum weekly rate series to identify epidemiologically anomalous seasons without imposing distributional assumptions [22]. Six features were derived for each season: peak weekly rate, mean weekly rate, cumulative rate, number of observed weeks, peak MMWR week, and coefficient of variation in weekly rates. The 2020–2021 season was excluded from the feature matrix due to CDC structural data suppression. Features were standardized using parameters derived from pre-pandemic seasons only, ensuring that the normative distribution reflected pre-pandemic burden. The primary model used 500 trees and a contamination parameter of 0.10. Robustness was assessed via a sensitivity sweep across contamination values of 0.05, 0.10, and 0.15; seasons flagged as anomalous across all three values were designated robust anomalies. Cross-method convergence between Prophet forecast gaps and Isolation Forest classifications was assessed for all seasons (Supplementary Materials S5).

### 2.12. Sensitivity Analyses and Inferential Considerations

Pre-specified a priori analyses included phase classification, denominator-version handling, the Isolation Forest contamination parameter (c = 0.10), the Prophet flat-versus-linear growth specification, and race and ethnicity rate-ratio estimation against the White reference. Robustness of the primary findings was evaluated through additional analyses (Supplementary Materials S4). The Isolation Forest classification was re-evaluated under three modifications: (1) exclusion of the season-length feature; (2) truncation of the 2023–2024 season at the standard 30-week surveillance window; and (3) leave-one-out refitting across pre-pandemic training seasons. The OLS phase regression was re-fit under the same truncation, and effect sizes for pairwise phase contrasts were estimated using Cohen’s d and Hedges’ g with bias-corrected and accelerated (BCa) bootstrap 95% confidence intervals. Hospitalization rates were also stratified descriptively by influenza type, A subtype, age group, and phase. Race and ethnicity rate ratios and pairwise phase contrasts were not adjusted for multiple comparisons; estimates are reported with bootstrap confidence intervals to convey precision rather than as null-hypothesis tests.

## 3. Results

### 3.1. Phase-Stratified Descriptive Findings

**Figure 1:**
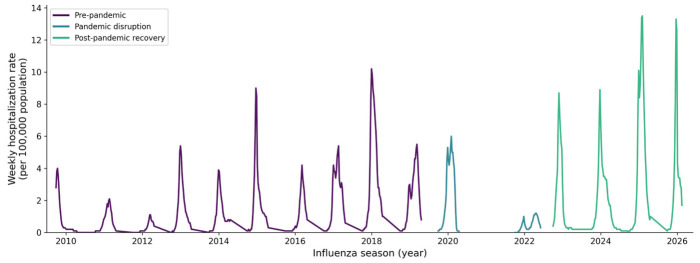
Weekly influenza hospitalization rates per 100,000 population, FluSurv-NET surveillance network, United States, 2009–2025.

The analytic dataset comprised 509 observation weeks across 16 complete influenza seasons (2009–2010 through 2024–2025); the in-progress 2025–2026 season is summarized descriptively only. Overall weekly hospitalization rates were broadly comparable between the pre-pandemic and pandemic disruption phases at the median level (0.8 and 0.6 per 100,000, respectively), while the post-pandemic recovery phase showed both higher median weekly rates (1.0 per 100,000) and substantially elevated peak and cumulative burden. Mean peak weekly rate nearly doubled from 5.1 per 100,000 (SD 2.8) in the pre-pandemic phase to 11.1 per 100,000 (SD 2.7) in recovery, and mean end-of-season cumulative rate increased from 46.3 to 87.0 per 100,000. Season timing shifted markedly, with median peak MMWR week advancing from week 9 in the pre-pandemic phase to week 50 in recovery, reflecting a transition from late-winter to early-winter seasonal peaks. Active season duration remained stable across all three phases (median 30 weeks). Full phase- and season-level descriptive statistics are presented in Table 1 (Figure 1).

**Figure 2:**
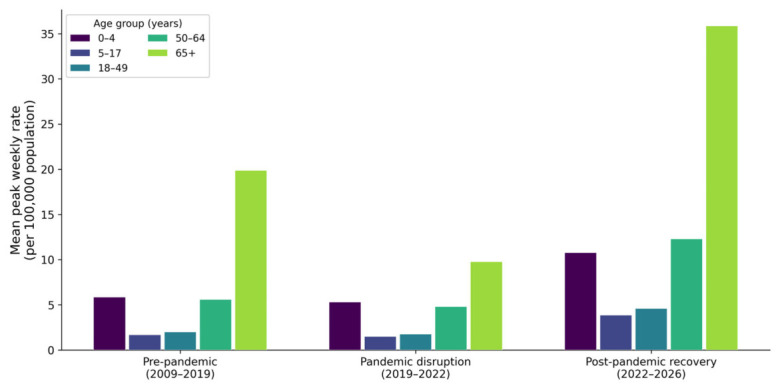
Mean peak weekly influenza hospitalization rates by age group and epidemiological phase, FluSurv-NET surveillance network, United States, 2009–2025.

By age group, adults aged 65 and older carried the highest burden across all three phases, with mean peak weekly rates of 19.9, 9.8, and 35.9 per 100,000 in the pre-pandemic, disruption, and recovery phases respectively—substantially exceeding all younger groups in every phase (Figure 2). Adults aged 50–64 showed the largest absolute increase in mean cumulative rate from pre-pandemic to recovery (50.8 to 96.7 per 100,000), followed by children aged 0–4 (53.6 to 88.8 per 100,000). The recovery-phase shift in peak timing to early winter was consistent across all age groups. Sex-stratified rates were similar between males and females within each phase, with females showing marginally higher mean cumulative rates in recovery (92.5 vs. 81.3 per 100,000). By virus type, Influenza A dominated all three phases and drove the majority of recovery-phase burden (mean cumulative rate 80.1 per 100,000), while Influenza B remained markedly suppressed through the disruption phase and into recovery (mean cumulative rate 5.8 per 100,000). Both A(H1N1)pdm09 and A(H3N2) subtypes showed recovery-phase resurgence with substantially shifted peak timing relative to pre-pandemic baselines.

### 3.2. Race/Ethnicity Disparities in Hospitalization Rates

**Figure 3:**
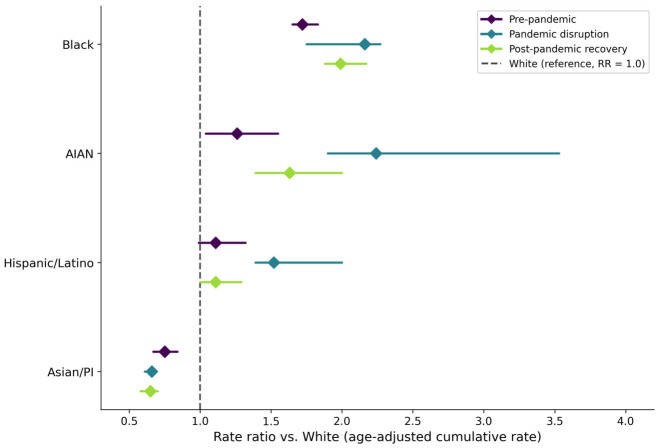
Age-adjusted influenza hospitalization rate ratios by race/ethnicity and epidemiological phase, FluSurv-NET surveillance network, United States, 2009–2025.

Racial and ethnic disparities in age-adjusted influenza hospitalization rates were present across all three epidemiological phases and persisted into the post-pandemic recovery period (Table 2; Figure 3). Black persons experienced the most consistently elevated burden relative to the White reference group, with rate ratios of 1.72 (95% CI 1.65–1.83), 2.16 (95% CI 1.75–2.27), and 1.99 (95% CI 1.88–2.17) in the pre-pandemic, disruption, and recovery phases, respectively. The disruption-phase elevation represents a widening of a pre-existing disparity that attenuated only partially in recovery. American Indian/Alaska Native persons had the highest disruption-phase rate ratio of any group examined (RR 2.24, 95% CI 1.90–3.53), though this estimate is based on two seasons with mixed denominator versions and carries substantial uncertainty as reflected in the wide confidence interval; the recovery-phase ratio attenuated to 1.63 (95% CI 1.39–2.00). Hispanic/Latino persons showed a pre-pandemic elevation with confidence intervals spanning the null (RR 1.11, 95% CI 0.99–1.32), a clearly elevated disruption-phase ratio (RR 1.52, 95% CI 1.39–2.00), and a return toward pre-pandemic levels in recovery (RR 1.11, 95% CI 1.00–1.29). Asian/Pacific Islander persons consistently showed rates below the White reference across all phases (RR 0.75, 0.66, and 0.65 in the pre-pandemic, disruption, and recovery phases, respectively); the narrow disruption-phase confidence interval (95% CI 0.61–0.67) reflects limited between-season variability in this group across the two available seasons rather than high estimation precision. Cross-phase comparisons of absolute adjusted rates should be interpreted with caution given the population denominator discontinuity at the 2020–2021 season boundary; within-phase rate ratios are internally consistent.

### 3.3. Phase-Level Differences in Mean Weekly Hospitalization Rates

Pairwise effect sizes indicated large differences between the recovery and both prior phases: Cohen’s d was 1.11 for pre-pandemic versus recovery and 0.97 for disruption versus recovery, with a small effect between pre-pandemic and disruption (d = 0.17), consistent with the descriptive similarity between those phases.

Season-level OLS regression estimated a pre-pandemic intercept of 1.51 per 100,000 (95% CI 0.80–2.22, *p* < 0.001). Recovery-phase coefficients were positive across both model specifications (primary: β = 1.13, 95% CI −0.35 to 2.60, *p* = 0.12; sensitivity: β = 2.76, 95% CI −0.85 to 6.37, *p* = 0.12) but did not reach statistical significance, reflecting substantial between-season variability at this sample size. The gap between unadjusted and adjusted R^2^ in the primary model (0.203 vs. 0.071) is consistent with limited degrees of freedom at *n* = 15 seasons rather than model misspecification, and effect size estimation is foregrounded accordingly. The disruption-phase coefficient was near zero and non-significant in both specifications (primary: β = −0.16, *p* = 0.84). The sensitivity model, which adjusted for population denominator version and the 2009–2010 pandemic season, produced directionally consistent estimates with reduced fit relative to the primary specification (adjusted R^2^ 0.057 vs. 0.071; Supplementary Materials S3).

A linear mixed-effects model with a season-level random intercept yielded a random effect variance of zero (ICC = 0), confirming that phase classification was determined entirely at the season level, which absorbed all between-season variance and precluded estimation of phase fixed effects. The within-season week coefficient was −0.055 per 100,000 per week (95% CI −0.074 to −0.037, *p* < 0.001), reflecting a consistent within-season decline from peak toward the inter-seasonal baseline (Supplementary Materials S3).

### 3.4. Structural Shift in Seasonality

**Figure 4:**
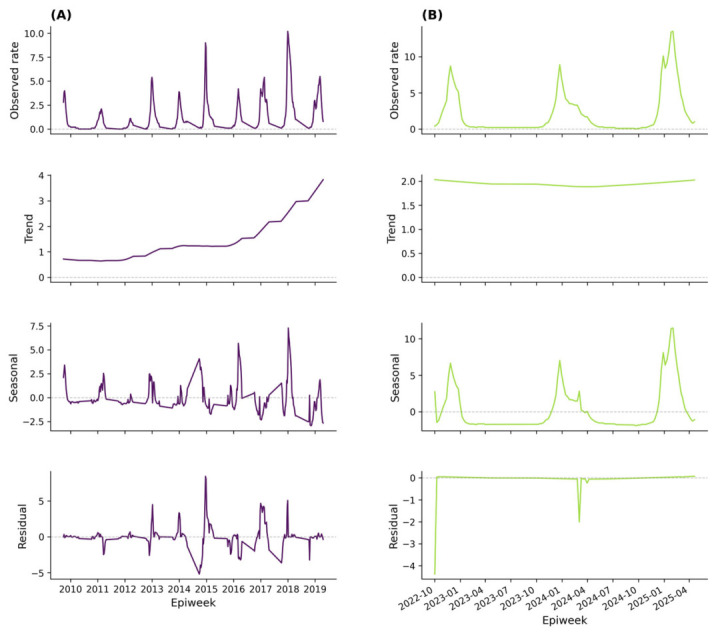
STL decomposition of weekly influenza hospitalization rates, FluSurv-NET, United States. (**A**): pre-pandemic series (2009–2010 to 2018–2019; *n* = 300 weeks). (**B**): post-pandemic recovery series (2022–2023 to 2024–2025; *n* = 112 weeks). Each panel shows the observed rate, extracted trend, seasonal, and residual components from top to bottom. Decomposition performed using robust LOESS with a 52-week period. All rates per 100,000 population. The pandemic disruption phase (2019–2020 to 2021–2022) was excluded from both series.

STL decomposition revealed a marked structural shift in the seasonal character of influenza hospitalization between the pre-pandemic and post-pandemic recovery periods (Figure 4). In the pre-pandemic series (300 weeks; ten seasons), seasonality was weak (Fs = 0.137) and the dominant source of variation was unstructured residual noise (SD 1.64 per 100,000), with a moderate upward trend (Ft = 0.237; trend range 3.18 per 100,000) and a seasonal amplitude IQR of 1.23 per 100,000 (Figure 4A). In the post-pandemic recovery series (112 weeks; three seasons), this structure was substantially reorganized: strength of seasonality increased to 0.979, residual SD collapsed to 0.45 per 100,000, and the trend component was effectively absent (Ft = 0.003; trend range 0.15 per 100,000), with seasonal amplitude nearly tripling to 3.44 per 100,000 (Figure 4B). These estimates should be interpreted with caution, given the limited series length, though the magnitude of the shift across all four decomposition metrics is consistent with a genuine structural change rather than an artifact of the shorter window. The recovery phase is thus characterized by a highly structured, repeating annual cycle with minimal residual noise and no discernible secular trend, in contrast to the heterogeneous, trend-driven pre-pandemic pattern.

### 3.5. Time-Series Forecasting

**Figure 5:**
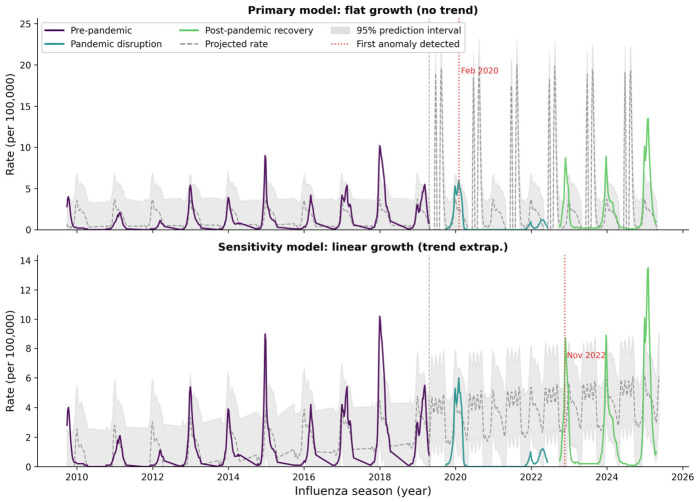
Prophet time-series forecast of weekly influenza hospitalization rates under primary and sensitivity specifications, FluSurv-NET, United States, 2009–2025. Both models were trained on pre-pandemic surveillance data (2009–2010 to 2018–2019; *n* = 300 weeks). The top panel shows the primary flat growth specification (no trend extrapolation); the bottom panel shows the sensitivity linear growth specification. The dashed line in each panel represents the model-projected rate; the shaded band represents the 95% prediction interval. Observed rates are shown by epidemiological phase (pre-pandemic, pandemic disruption, post-pandemic recovery). The vertical dashed line indicates the training cutoff (April 2019); the vertical dotted line indicates the first week in which observed rates exceeded the upper bound of the 95% prediction interval (February 2020 for the flat growth model; November 2022 for the linear growth model). The 146-week divergence in first detection date reflects systematic over-projection by the linear model in the post-pandemic period. All rates per 100,000 population. See Supplementary Materials S4 for held-out validation metrics.

A Prophet model trained on 300 weeks of pre-pandemic surveillance data (2009–2010 to 2018–2019) was used to generate a counterfactual baseline representing expected hospitalization rates’ absent pandemic disruption (Figure 5). The primary specification used a flat growth assumption, holding the pre-pandemic rate level constant without trend extrapolation. Under this model, the first exceedance of the 95% prediction interval upper bound occurred in early February 2020, coinciding with the onset of pandemic-period disruption. The February 2020 detection date was identical across 9 of 10 leave-one-out refits excluding individual training seasons, with the sole exception advancing detection by 2 weeks, confirming the finding is not attributable to any single pre-pandemic season (Supplementary Materials S5). At the season level, mean observed rates in the recovery phase exceeded the flat model projection across all three complete recovery seasons, with the largest mean gap in 2024–2025 (observed 4.21 vs. projected 1.53 per 100,000; gap +2.68) and more modest exceedances in 2022–2023 and 2023–2024 (gaps +0.56 and +0.54 per 100,000, respectively). The disruption-phase gap was mixed: 2019–2020 showed a modest positive mean gap (+0.67 per 100,000), while 2021–2022 showed a large positive gap (+1.35 per 100,000), reflecting a projected baseline held at pre-pandemic levels against near-zero observed rates during the pandemic-associated collapse of influenza activity that season (Supplementary Materials S4).

A sensitivity model using linear growth extrapolated the pre-pandemic upward trend into the forecast period, substantially elevating the projected baseline over time. Under this specification, first exceedance was delayed to November 2022, 146 weeks later than the flat model and well exceeding the pre-specified four-week convergence threshold. The linear model systematically over-projected mean rates in all recovery seasons (projected means 3.53 to 4.37 per 100,000 vs. observed 1.61 to 4.21 per 100,000), attributing observed burden to continued trend rather than anomalous elevation. Peak-level gap comparisons under the linear model are not reported for 2023–2024, as the projected peak for that season reflects a forecasting artifact driven by its atypical 52-week duration rather than a meaningful model estimate. Given the flat model’s substantially superior held-out predictive performance in the 2018–2019 validation season (MAE 1.01 vs. 1.59; R^2^ 0.23 vs. 0.005), the linear model’s delayed detection reflects over-projection from trend extrapolation rather than a later true onset of anomalous burden. Model growth specification sensitivity is outlined in Supplementary Materials S4.

### 3.6. Robust Anomalous Seasons

**Figure 6:**
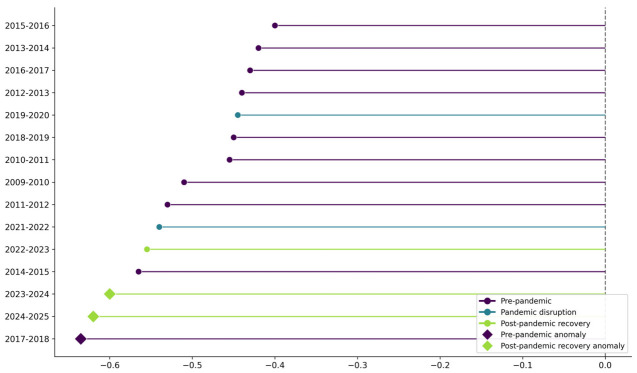
Isolation Forest anomaly scores by season, FluSurv-NET, United States, 2009–2025. Seasons are ranked by anomaly score from most to least anomalous (top to bottom). More negative scores indicate greater departure from the pre-pandemic normative distribution. Diamond markers indicate seasons classified as anomalous at the primary contamination threshold (0.10); circle markers indicate seasons classified as normal. Colors correspond to epidemiological phase. The 2020–2021 season was excluded from scoring due to CDC structural suppression of weekly hospitalization data. Model trained on pre-pandemic seasons (2009–2010 to 2018–2019) with standardized features: peak rate, mean rate, cumulative rate, season duration, peak week, and rate coefficient of variation. See Supplementary Materials S4 for full feature matrix and contamination sensitivity sweep.

The 2020–2021 season was excluded from Isolation Forest scoring due to CDC structural suppression of weekly hospitalization data during that period. Of the remaining 15 seasons, three were classified as robust anomalies, defined as flagged at all three contamination thresholds in the sensitivity sweep (0.05, 0.10, and 0.15): 2017–2018, 2023–2024, and 2024–2025 (Figure 6; Supplementary Materials S5). The model was trained on pre-pandemic seasons (2009–2010 to 2018–2019) using six standardized season-level features: peak rate, mean rate, cumulative rate, season duration, peak week, and rate coefficient of variation (Supplementary Materials S4).

The 2017–2018 pre-pandemic season received the most anomalous score in the dataset (−0.623), distinguished by the highest pre-pandemic peak rate (10.2 per 100,000), mean weekly rate (3.42 per 100,000), and cumulative rate (102.7 per 100,000) in the training distribution, with peak burden concentrated at MMWR week 1. The 2024–2025 and 2023–2024 seasons ranked second and third in anomaly score (−0.615 and −0.600, respectively), with peak rates of 13.5 and 8.9 per 100,000 and cumulative rates of 126.3 and 83.7 per 100,000, each exceeding the pre-pandemic normative distribution across multiple feature dimensions. The 2014–2015 season was flagged only at the highest contamination threshold (0.15) and is not considered a robust anomaly. All three robust anomalies showed concordant classification across both methods, with positive Prophet forecast gaps and Isolation Forest anomaly flags in each case, providing cross-method validation of the primary finding (Supplementary Materials S4).

## 4. Discussion

Post-pandemic influenza in the United States does not represent a simple return to pre-pandemic norms. The recovery period captured through FluSurv-NET surveillance is instead characterized by compressed but substantially intensified seasonality, elevated hospitalization burden across demographic groups, and persistent racial and ethnic disparities that widened during the pandemic disruption period and have not fully resolved. These findings, derived retrospectively from a decade of population-based surveillance data using time-series decomposition, counterfactual forecasting, and unsupervised anomaly detection, are mutually reinforcing and collectively suggest that the structural character of influenza seasonality in the United States has undergone a meaningful reorganization in the post-pandemic period.

The burden elevation observed in the recovery phase is consistent with the immunological debt hypothesis, which holds that suppression of influenza transmission during the pandemic period through non-pharmaceutical interventions reduced opportunities for natural immune boosting at the population level, producing an expanded susceptible cohort when community mixing resumed [4,5,6]. This rebound was not uniform across influenza types or age strata: influenza A drove the recovery-phase resurgence (mean cumulative rate rising from 38.2 to 80.1 per 100,000) while influenza B remained at pre-pandemic levels, and within influenza A, A(H1N1)pdm09 showed the largest proportional rebound. The age-stratified pattern further suggests subtype-specific susceptibility dynamics, with school-aged children (5–17 years) showing the largest proportional rebound and adults aged 65 and older bearing the largest absolute burden, consistent with cohort-specific immunological naïveté among younger populations and waning subtype-specific immunity among older adults in the absence of seasonal boosting between 2020 and 2022. Adults aged 65 and older carried the highest absolute burden across all three phases, and the post-pandemic amplification of that burden is epidemiologically consequential given the well-established relationship between influenza severity and age-related immune senescence [23,24,25]. The dramatic advance in median peak timing from late winter to early winter across all age groups and virus types adds a planning dimension to the burden finding: vaccination program schedules and healthcare surge frameworks calibrated to historical late-season peaks may be poorly aligned with the current seasonal pattern, and prospective monitoring is warranted to determine whether early-winter onset is a durable feature of the post-pandemic landscape or a transitional phenomenon [7,9,26].

The STL decomposition results provide quantitative support for a structural change in seasonal influenza dynamics that extends beyond burden elevation alone. Pre-pandemic influenza activity was dominated by residual noise, with weak and irregular seasonality consistent with heterogeneous epidemic dynamics driven by shifting antigenic profiles and variable population immunity across seasons. The post-pandemic recovery period presents a qualitatively different picture: near-perfect seasonal regularity, negligible residual variance, and no discernible secular trend. This transition from a noise-dominated to a seasonality-dominated signal is epidemiologically interpretable. Pandemic-period suppression of influenza circulation likely produced a susceptible population with reduced prior-season immunity heterogeneity, enabling more uniform and temporally predictable seasonal dynamics upon re-emergence [27,28,29]. From a preparedness standpoint, higher-amplitude but more predictable annual cycles could support more precise surge planning and resource allocation if the pattern persists. Whether this regularity reflects a durable post-pandemic immune landscape shift or a transitional period remains an open question; the recovery-phase observation window spans only three complete seasons, and between-season variability in vaccine composition, uptake, and antigenic match will continue to influence the degree of structural regularity observed in future seasons.

The racial and ethnic disparity findings extend a well-documented pattern of inequitable influenza burden with new post-pandemic context [10,11,30]. Black persons experienced the highest and most consistently elevated hospitalization rates relative to the White reference group across all three phases. The disparity widened during the disruption period and attenuated only partially in recovery, consistent with longstanding racial disparities in infectious disease hospitalization burden documented across seasons in the United States [10,31]. American Indian and Alaska Native (AI/AN) persons showed the largest disruption-phase elevation of any group examined, though this estimate carries substantial uncertainty reflecting the limited number of contributing seasons. This pattern is consistent with documented disparities in infectious disease outcomes among Indigenous populations in the United States [32,33] and internationally, reflecting systemic rather than incidental inequities in burden distribution [34]. The disruption-phase widening for these groups maps onto structural conditions that became acutely visible during the COVID-19 period: populations with constrained ability to reduce occupational exposure, access timely care, or benefit consistently from vaccination programs faced compounded infectious disease burden when influenza co-circulated with SARS-CoV-2 [35,36]. The partial return of Hispanic and Latino rates toward pre-pandemic levels in recovery, in contrast to the persistence of elevated Black and AI/AN rates, suggests heterogeneity in disparity mechanisms and trajectories across groups that aggregate rate ratios cannot fully characterize. The present analysis is descriptive and does not adjust for vaccination status, comorbidity burden, or healthcare access; the rate ratios reported here reflect population-level burden distribution rather than etiologic attribution.

The convergent findings from Prophet forecasting and Isolation Forest anomaly detection strengthen the inferential case for recovery-phase burden elevation beyond what descriptive statistics alone could support. The two methods approach the problem differently: Prophet evaluates week-level exceedance of a pre-pandemic prediction interval, while Isolation Forest scores season-level feature vectors against a pre-pandemic normative distribution. Both independently identified the 2023–2024 and 2024–2025 seasons as anomalous. The consistent flagging of the 2017–2018 season as the sole pre-pandemic anomaly by both methods provides internal validation, confirming sensitivity to genuine epidemiological outliers rather than recovery-phase artifacts; that season was previously characterized in the literature as unusually severe and was associated with predominant A(H3N2) circulation [37]. The 146-week divergence in first detection date between flat and linear growth Prophet specifications illustrates the degree to which surveillance-oriented forecasting is sensitive to baseline trend assumptions, and underscores the importance of prospective model validation before deployment in operational settings.

Several limitations of this analysis warrant acknowledgment. FluSurv-NET covers approximately 10% of the US population across 14 states and is not designed to produce nationally representative rate estimates; regional heterogeneity in influenza burden remains uncharacterized. Within-state catchment coverage varies substantially across participating sites, ranging from approximately 9% to 71% of state-level population; within-state representativeness is therefore not uniform, and state-level rate comparisons were not pursued. The season-level OLS regression is based on 15 seasons and is underpowered to detect modest phase differences; effect size estimates are foregrounded over *p*-values accordingly. The disparity analysis presents descriptive age-adjusted rate ratios and does not adjust for vaccination status, comorbidity prevalence, or healthcare access, and should not be interpreted as an etiologic analysis of disparity mechanisms. Relatedly, this analysis was restricted to laboratory-confirmed hospitalization rates and did not incorporate measures of disease severity. FluSurv-NET captures intensive care unit admission, mechanical ventilation, and in-hospital and post-discharge mortality through sampled chart abstractions with variable sampling fractions across seasons, and valid phase-stratified severity comparisons would require selection-probability weighting beyond the scope of the present descriptive analysis. Similarly, this analysis did not integrate season-specific influenza vaccination coverage or vaccine effectiveness estimates, which vary by age, region, and circulating subtype and declined during and after the COVID-19 pandemic; vaccination dynamics could plausibly modify the magnitude of recovery-phase rate elevations and represent an important target for future integrative analyses. The population denominator transition from bridged to unbridged race and ethnicity estimates at the 2020–2021 boundary limits direct cross-phase comparison of absolute rates, though within-phase rate ratios are internally consistent. The Isolation Forest model was trained on ten pre-pandemic seasons, and the small training set limits threshold precision; contamination sensitivity analyses were conducted to address this constraint, and findings were restricted to seasons flagged robustly across all levels. Subgroup-level anomaly detection stratified by age or race and ethnicity was outside the scope of the present analysis and represents a direction for future work.

## 5. Conclusions

Sixteen complete seasons of FluSurv-NET surveillance data demonstrate a post-pandemic influenza recovery characterized by substantially elevated hospitalization burden, a structural shift toward compressed and highly regular annual seasonality, earlier-onset seasonal peaks, and anomalous severity in the two most recent complete seasons identified independently by time-series forecasting and unsupervised anomaly detection. Racial and ethnic disparities in hospitalization rates were substantial prior to the pandemic, widened during the disruption period, and have not returned to pre-pandemic levels for several groups, reflecting heterogeneity in underlying population risk that aggregate burden estimates alone do not capture. Whether the elevated and reorganized recovery-phase pattern represents a transient resurgence or a more durable shift in the influenza epidemiological baseline cannot be determined from three recovery seasons alone. The analytic approach applied here, combining decomposition, counterfactual forecasting, and anomaly detection against a pre-pandemic normative baseline, is applicable to other population-based surveillance systems navigating similar post-pandemic questions. Continued surveillance with standardized methodology across future seasons remains essential for characterizing the trajectory of influenza burden and informing preparedness planning in the post-pandemic era.

## Figures and Tables

**Table 1:** Phase-stratified influenza hospitalization rates by demographic and virologic subgroup, FluSurv-NET, United States, 2009–2025.

Group	Median Weekly Rate PRE (IQR)	Median Weekly Rate DISR (IQR)	Median Weekly Rate REC (IQR)	Mean Peak Rate PRE (SD)	Mean Peak Rate DISR (SD)	Mean Peak Rate REC (SD)	Median Peak Week PRE (IQR)	Median Peak Week DISR (IQR)	Median Peak Week REC (IQR)	Mean Cumulative Rate PRE (SD)	Mean Cumulative Rate DISR (SD)	Mean Cumulative Rate REC (SD)	Median Season Duration PRE (IQR)	Median Season Duration DISR (IQR)	Median Season Duration REC (IQR)
Panel A. Overall															
Overall	0.8 (0.2–2.2)	0.6 (0.2–1.2)	1.0 (0.2–3.5)	5.1 (2.8)	3.6 (3.4)	11.1 (2.7)	9 (3–11)	11 (8–14)	50 (38–52)	46.3 (27.4)	28.1 (33.9)	87.0 (27.5)	30 (28–30)	30 (30–30)	30 (28–35)
Panel B. Age group															
0–4	1.0 (0.3–2.6)	0.8 (0.2–1.6)	1.1 (0.2–3.9)	5.8 (2.3)	5.3 (5.2)	10.8 (2.6)	9 (5–34)	10 (5–14)	50 (37–52)	53.6 (18.7)	57.0 (48.7)	88.8 (10.6)	30 (28–30)	30 (29–32)	30 (27–34)
5–17	0.3 (0.1–0.6)	0.3 (0.1–0.6)	0.6 (0.1–1.7)	1.7 (1.1)	1.5 (1.1)	3.9 (0.9)	10 (7–34)	11 (8–14)	49 (37–51)	14.6 (6.8)	16.1 (10.2)	33.8 (4.7)	24 (21–26)	26 (25–27)	28 (25–31)
18–49	0.3 (0.1–0.9)	0.3 (0.1–0.6)	0.5 (0.1–1.6)	2.0 (0.8)	1.8 (1.6)	4.6 (0.8)	8 (3–11)	11 (8–14)	50 (38–52)	18.4 (7.2)	21.2 (17.0)	36.6 (10.1)	26 (25–28)	28 (27–29)	30 (27–32)
50–64	0.8 (0.2–2.5)	0.5 (0.2–1.2)	1.1 (0.3–4.1)	5.6 (2.8)	4.8 (5.1)	12.3 (3.0)	9 (4–12)	12 (9–14)	50 (38–52)	50.8 (29.8)	52.8 (51.8)	96.7 (39.3)	30 (27–30)	30 (30–30)	30 (28–35)
65+	2.4 (0.4–6.9)	1.4 (0.5–3.4)	3.0 (0.7–10.6)	19.9 (16.5)	9.8 (8.4)	35.9 (11.7)	9 (2–11)	12 (9–14)	50 (37–52)	170.6 (137.3)	111.6 (85.7)	263.1 (92.0)	30 (30–30)	32 (31–32)	30 (28–35)
Panel C. Sex															
Male	0.7 (0.2–2.2)	0.6 (0.2–1.1)	1.1 (0.2–3.4)	4.8 (2.6)	3.5 (3.5)	10.2 (2.5)	9 (3–11)	12 (9–14)	50 (37–52)	43.6 (25.3)	40.1 (33.9)	81.3 (26.6)	30 (28–30)	30 (30–30)	30 (28–35)
Female	0.8 (0.2–2.5)	0.6 (0.2–1.3)	1.1 (0.3–3.8)	5.4 (3.0)	3.7 (3.4)	11.9 (2.8)	9 (3–12)	11 (8–14)	50 (38–52)	48.8 (29.4)	43.4 (34.6)	92.5 (28.6)	28 (28–30)	30 (29–31)	30 (28–35)
Panel D. Virus type															
Influenza A	0.5 (0.1–1.8)	0.5 (0.2–1.1)	0.9 (0.2–3.2)	4.7 (2.6)	3.0 (2.5)	10.7 (2.9)	8 (2–11)	11 (8–14)	50 (38–52)	38.2 (20.9)	21.7 (24.2)	80.1 (27.7)	28 (26–30)	29 (28–30)	30 (28–35)
Influenza B	0.1 (0.0–0.3)	0.0 (0.0–0.3)	0.1 (0.0–0.2)	0.7 (0.7)	1.1 (1.6)	0.5 (0.3)	10 (8–12)	1 (1–1)	4 (1–8)	7.8 (8.1)	6.2 (9.9)	5.8 (4.4)	21 (18–24)	12 (6–18)	22 (19–25)
A(H1N1)pdm09	0.1 (0.0–0.3)	0.0 (0.0–0.4)	0.3 (0.1–1.5)	1.3 (1.4)	2.3 (3.2)	5.7 (3.4)	7 (2–8)	4 (2–5)	48 (27–50)	11.5 (12.2)	22.9 (32.3)	44.7 (29.0)	14 (7–20)	11 (6–16)	25 (25–28)
A(H3N2)	0.2 (0.1–0.9)	0.2 (0.0–0.4)	0.3 (0.0–1.3)	3.5 (3.2)	0.7 (0.7)	4.5 (2.6)	10 (7–11)	34 (26–43)	5 (3–26)	27.8 (23.1)	10.0 (10.6)	38.6 (14.8)	21 (19–25)	21 (17–25)	25 (22–26)
Panel E. Race/ethnicity															
White	0.7 (0.1–2.0)	0.4 (0.2–1.1)	1.0 (0.2–3.5)	4.9 (3.1)	3.2 (2.9)	11.1 (3.3)	9 (2–11)	11 (8–14)	50 (37–52)	43.3 (29.1)	36.1 (29.0)	85.3 (30.8)	28 (26–30)	30 (30–30)	30 (28–35)
Black	1.0 (0.2–2.8)	0.7 (0.3–1.7)	1.6 (0.4–5.5)	6.5 (3.4)	5.5 (5.8)	18.5 (5.7)	10 (8–34)	10 (8–12)	50 (38–52)	57.6 (31.1)	64.3 (58.5)	134.4 (40.3)	30 (28–30)	30 (30–31)	30 (28–35)
Hispanic/Latino	0.5 (0.1–1.7)	0.6 (0.2–1.5)	0.9 (0.2–2.6)	3.4 (1.4)	3.6 (2.8)	8.0 (1.0)	9 (1–33)	8 (4–11)	50 (36–52)	31.8 (13.6)	38.7 (24.7)	64.2 (12.7)	28 (26–29)	29 (28–30)	29 (27–33)
Asian/Pacific Islander	0.4 (0.1–1.2)	0.3 (0.1–0.5)	0.6 (0.2–1.7)	3.0 (1.9)	1.6 (1.6)	4.8 (1.0)	9 (1–11)	10 (7–12)	24 (1–49)	25.9 (16.0)	18.8 (15.7)	42.9 (17.5)	26 (25–28)	25 (25–25)	29 (27–34)
American Indian/Alaska Native	0.5 (0.0–2.0)	1.0 (0.0–3.4)	1.2 (0.0–5.0)	5.8 (2.3)	7.8 (4.7)	14.4 (2.0)	6 (2–10)	10 (6–14)	50 (37–51)	43.6 (21.5)	68.6 (32.7)	114.8 (35.6)	21 (18–21)	23 (22–24)	22 (19–27)

All rates are per 100,000 population. Background shading denotes study phase: pre-COVID-19 pandemic (blue), COVID-19 pandemic disruption (orange), post-disruption recovery (green). Phase abbreviations: PRE = pre-pandemic baseline (2009–2010 to 2018–2019); DISR = pandemic disruption (2019–2020 to 2021–2022); REC = post-pandemic recovery (2022–2023 to 2024–2025). Weekly rate summaries exclude the 2020–2021 season, during which CDC structurally suppressed weekly hospitalization data; cumulative rates for 2020–2021 are retained where available. Peak week is reported as MMWR epidemiological week number. Season duration reflects the number of weeks with a weekly hospitalization rate ≥ 0.1 per 100,000. IQR = interquartile range; SD = standard deviation.

**Table 2:** Age-adjusted influenza hospitalization rate ratios by race/ethnicity and epidemiological phase, FluSurv-NET, United States, 2009–2025.

Phase	Race/Ethnicity Group	Seasons (n)	Mean Age-Adjusted Rate (per 100,000)	Rate Ratio	95% CI	Denominator Note
PRE						
	White (reference)	10	35.74	1	Reference	bridged
	Black	10	61.56	1.72	1.65–1.83	bridged
	Hispanic/Latino	10	39.8	1.11	0.99–1.32	bridged
	Asian/Pacific Islander	10	26.65	0.75	0.67–0.84	bridged
	American Indian/Alaska Native	10	45.15	1.26	1.04–1.55	bridged
DISR						
	White (reference)	2	32.39	1	Reference	bridged, unbridged
	Black	2	70.06	2.16	1.75–2.27	bridged, unbridged
	Hispanic/Latino	2	49.28	1.52	1.39–2.0	bridged, unbridged
	Asian/Pacific Islander	2	21.3	0.66	0.61–0.67	bridged, unbridged
	American Indian/Alaska Native	2	72.7	2.24	1.9–3.53	bridged, unbridged
REC						
	White (reference)	4	73.89	1	Reference	unbridged
	Black	4	146.73	1.99	1.88–2.17	unbridged
	Hispanic/Latino	4	82.24	1.11	1.0–1.29	unbridged
	Asian/Pacific Islander	4	47.72	0.65	0.58–0.7	unbridged
	American Indian/Alaska Native	4	120.59	1.63	1.39–2.0	unbridged

Rate ratios compare age-adjusted end-of-season cumulative hospitalization rates for each racial/ethnic group relative to the Non-Hispanic White reference group. Phase abbreviations: PRE = pre-pandemic baseline (2009–2010 to 2018–2019); DISR = pandemic disruption (2019–2020 to 2021–2022); REC = post-pandemic recovery (2022–2023 to 2024–2025). The 2020–2021 season was excluded from all race-stratified analyses due to structural CDC suppression of race-specific hospitalization data. 95% confidence intervals derived by nonparametric bootstrap resampling of season-level pairs (1000 iterations, percentile method). Cross-phase comparisons of absolute rate levels should be interpreted with caution due to a denominator discontinuity between bridged (PRE) and unbridged (DISR, REC) population estimates; within-phase rate ratios are internally consistent. CI = confidence interval.

## Data Availability

All data analyzed in this study are publicly available. Influenza hospitalization surveillance data were obtained from the CDC FluSurv-NET Influenza Hospitalization Surveillance Network and are accessible through the CDC FluView Interactive platform at https://www.cdc.gov/fluview/overview/influenza-hospitalization-surveillance.html (accessed on 8 January 2025).

## Supplementary Materials

The following supporting information can be downloaded at: https://www.mdpi.com/article/10.3390/idr18030061/s1. Supplemental Materials S1, Analysis code and reproducibility (GitHub repository); Supplemental Materials S2, Descriptive and season-level detail (Table S1; Figure S1); Supplemental Materials S3, Phase-level statistical models (Tables S2 and S3); Supplemental Materials S4, Time-series forecasting and anomaly detection (Table S4; Figure S2); Supplemental Materials S5, Isolation Forest sensitivity analyses (Tables S5–S8; Figure S3).
