# Supplementary material for: Game Over for the Baseline: Influenza Hospitalization Patterns Before, During, and After the COVID-19 Pandemic (FluSurv-NET, 2009–2025)
Source: Infect Dis Rep. 2026 Jun 19;18(3):61. doi: 10.3390/idr18030061 (PMC13300026; doi:10.3390/idr18030061)
Supplement: Supplementary file 1 [file idr-18-00061-s001.zip › idr-4230991-supplementary.pdf]

Supplemental Materials S1 – Analysis Code and Reproducibility

The code used for this study is available in the public repository at: https://github.com/h-hedman/healthcare-data-science/tree/main/fluserv-net-surveillance.

All scripts required to reproduce the models, tables, and figures are provided.

Supplemental Materials S2 – Descriptive and Season-Level Detail

Table S1. Season-level influenza hospitalization descriptive statistics, FluSurv-NET: 2009–2025.

Season	Phase	Denom	Partial	H1N1 Season	Weeks (n)	Active Weeks (n)	Median Rate	Q1	Q3	Mean Rate	SD	Peak Rate	Peak Week	EOS Cum Rate	EOS Age-Adj Rate
2009-10	PRE	bridged	0	1	30	28	0.2	0.2	0.73	0.83	1.23	4	42	29.3	25.75
2010-11	PRE	bridged	0	0	30	29	0.45	0.1	1.2	0.72	0.67	2.1	8	21.5	19.76
2011-12	PRE	bridged	0	0	30	20	0.1	0	0.55	0.29	0.35	1.1	11	8.7	7.65
2012-13	PRE	bridged	0	0	30	27	0.85	0.22	1.98	1.47	1.6	5.4	1	44	40.86
2013-14	PRE	bridged	0	0	30	29	0.8	0.45	1.68	1.17	1.12	3.9	1	35.1	32.4
2014-15	PRE	bridged	0	0	30	30	1.2	0.35	2.45	1.93	2.32	9	52	64.1	55.86
2015-16	PRE	bridged	0	0	30	30	0.4	0.1	1.78	1.05	1.23	4.2	10	31.4	28.54
2016-17	PRE	bridged	0	0	30	30	1.8	0.32	3.38	2.07	1.75	5.4	8	62	56.16
2017-18	PRE	bridged	0	0	30	30	2.25	0.85	6.02	3.42	3.33	10.2	1	102.9	70.71
2018-19	PRE	bridged	0	0	30	30	2	0.38	3.38	2.12	1.75	5.5	11	63.6	59.99
2019-20	DISR	bridged	0	0	30	29	1.3	0.2	4.47	2.2	2.15	6	6	66	62.43
2020-21	DISR	unbridged	0	0	31	0							1	0.8	0.77
2021-22	DISR	unbridged	0	0	36	31	0.4	0.2	0.72	0.49	0.38	1.2	16	17.5	16.62
2022-23	REC	unbridged	0	0	30	30	0.55	0.3	3.15	2.08	2.6	8.7	48	62.4	59.13
2023-24	REC	unbridged	0	0	52	51	0.5	0.2	2.75	1.61	2.06	8.9	52	83.5	80.2

2024-25	REC	unbridged	0	0	30	30	2.05	0.52	8.22	4.21	4.43	13.5	6	126.2	121.95
2025-26	REC	unbridged	1	0	30	21	2.8	0.4	3.5	3.27	3.79	13.3	52	76	71.77

Season-level influenza hospitalization summary statistics for the overall FluSurv-NET surveillance network, 2009–2025. One row per season. All rates per 100,000 population. Phase abbreviations: PRE = pre-pandemic baseline (2009–10 to 2018–19); DISR = pandemic disruption (2019–20 to 2021–22); REC = post-pandemic recovery (2022–23 to 2024–25). Active weeks defined as weeks with a weekly hospitalization rate ≥0.1 per 100,000. The 2020–21 season median, mean, peak, and Q1/Q3 fields are blank due to structural CDC suppression of weekly hospitalization data; end-of-season cumulative rate is available for that season. Denom = population denominator version (bridged = pre-2020 bridged race estimates; unbridged = post-2020 unbridged estimates). Partial = partial season flag (1 = incomplete season at time of analysis). EOS = end of season. Q1/Q3 = 25th/75th percentile of weekly rates.

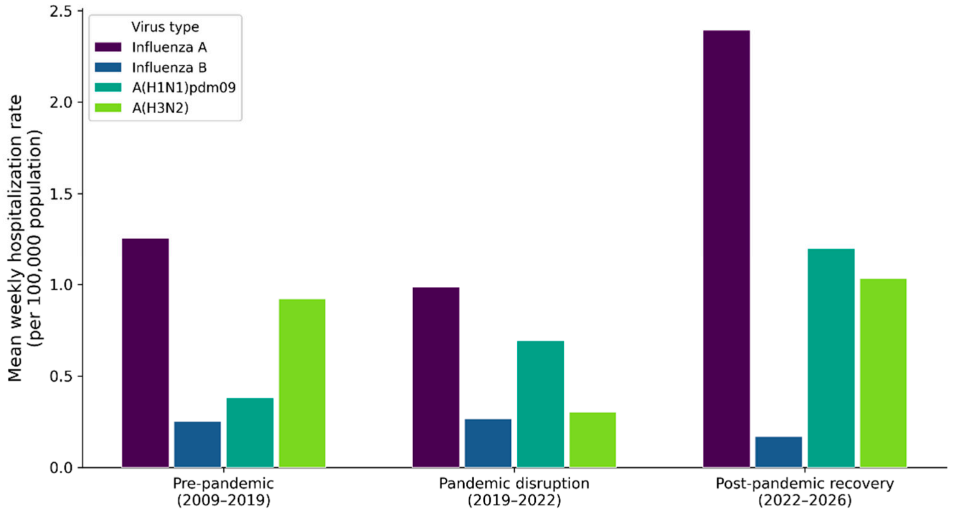

Figure S1. Season-level influenza hospitalization trajectories by virus type and epidemiological phase.

Supplemental Materials S3 – Phase-Level Statistical Models

Table S2. Ordinary least squares regression of mean weekly influenza hospitalization rate on epidemiological phase: primary and sensitivity models, FluSurv-NET, United States, 2009–2025.

**Model**	**Parameter**	**Estimate**	**SE**	**95% CI Lower**	**95% CI Upper**	**t**	**p**	**R^2**	**Adj R^2**	**Seasons (n)**
Primary	Intercept (pre-pandemic reference)	1.507	0.325	0.798	2.216	4.631	0.0006	0.203	0.071	15
Primary	Pandemic disruption vs pre-pandemic (DISR)	-0.164	0.797	-1.901	1.573	-0.206	0.8402	0.203	0.071	15
Primary	Post-pandemic recovery vs pre-pandemic (REC)	1.127	0.677	-0.349	2.603	1.664	0.122	0.203	0.071	15
Sensitivity	Intercept (pre-pandemic reference)	1.582	0.346	0.812	2.352	4.578	0.001	0.326	0.057	15
Sensitivity	Pandemic disruption vs pre-pandemic (DISR)	0.615	1.093	-1.82	3.05	0.563	0.5861	0.326	0.057	15
Sensitivity	Post-pandemic recovery vs pre-pandemic (REC)	2.76	1.621	-0.851	6.372	1.703	0.1194	0.326	0.057	15
Sensitivity	Denominator version (unbridged vs bridged)	-1.708	1.466	-4.974	1.559	-1.165	0.2711	0.326	0.057	15
Sensitivity	2009–10 H1N1 pandemic season indicator	-0.749	1.093	-3.183	1.686	-0.685	0.5089	0.326	0.057	15

Table S3. Linear mixed-effects model fixed effects within-season weekly influenza hospitalization rate trend, FluSurv-NET, United States, 2009–2025.

**Parameter**	**Estimate**	**SE**	**95% CI Lower**	**95% CI Upper**	**Seasons (n)**
Intercept (pre-pandemic reference)	0	10820536.78	-21207862.38	21207862.38	15
Within-season week number (linear trend)	-0.055	0.009	-0.074	-0.037	15

Intercept confidence interval reflects degenerate random-effect variance (ICC = 0); the fixed intercept is not interpretable.

Supplemental Materials S4 – Time-Series Forecasting and Anomaly Detection

Table S4. Prophet held-out validation metrics: flat vs linear growth specification

Model	MAR	RMSE	MAPE (%)	R^2	Test Weeks (n)
Prophet_linear	1.5897	1.7158	—	0.0053	30
Prophet_flat	1.0064	1.5079	86.14	0.2317	30

MAPE not reported for the linear growth model; unstable due to trend extrapolation over near-zero inter-seasonal observations.

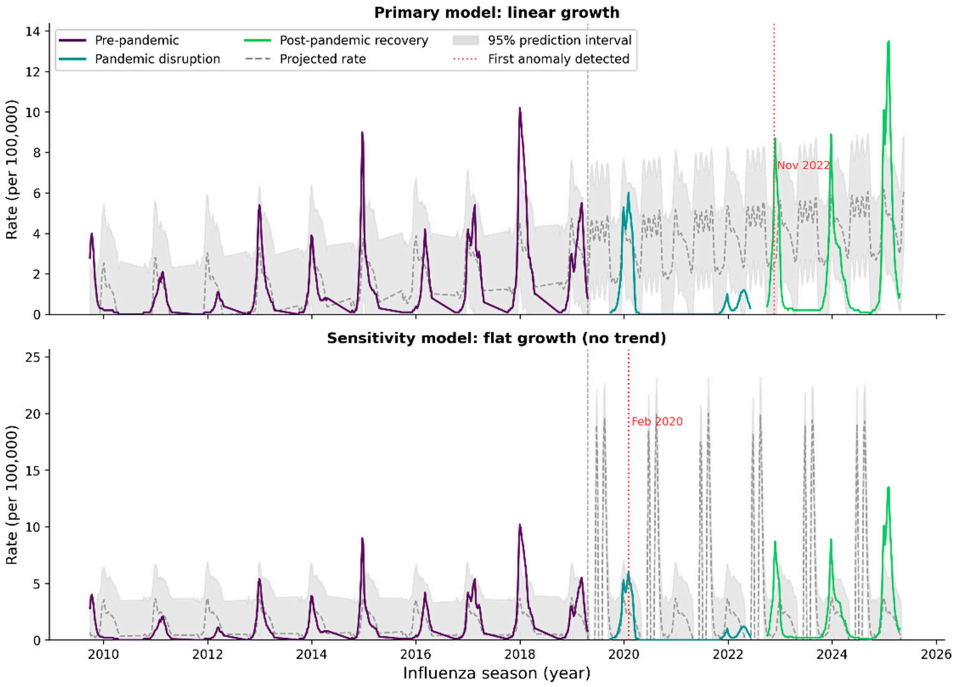

Figure S2. Prophet sensitivity model comparison: linear vs flat growth trend specification

Supplemental Materials S5 – Isolation Forest Sensitivity Analyses

The Isolation Forest model was trained on pre-pandemic seasons (2009–10 to 2018–19) to establish a normative burden distribution, with scoring applied to all remaining seasons. The 2020–21 season was excluded from both training and scoring; CDC-suppressed near-zero rates during that period reflect a reporting artifact rather than true disease burden, and inclusion would produce a misleadingly low anomaly score.

Table S5. Isolation Forest season-level feature matrix and anomaly classifications, FluSurv-NET, United States, 2009–2025 (2020–21 excluded).

Season	Phase	Rank	Classification	Anomaly Score	Peak Rate	Mean Rate	Cum Rate	Weeks (n)	Peak Week	Rate CV
2017-18	PRE	1	Anomaly	-0.623	10.2	3.423	102.7	30	1	0.972
2024-25	REC	2	Anomaly	-0.615	13.5	4.21	126.3	30	6	1.051
2023-24	REC	3	Anomaly	-0.6	8.9	1.61	83.7	52	52	1.277
2014-15	PRE	4	Normal	-0.569	9	1.93	57.9	30	30	1.2
2022-23	REC	5	Normal	-0.553	8.7	2.083	62.5	30	26	1.247
2021-22	DISR	6	Normal	-0.538	1.2	0.489	17.6	36	16	0.787
2011-12	PRE	7	Normal	-0.529	1.1	0.287	8.6	30	11	1.211
2009-10	PRE	8	Normal	-0.516	4	0.833	25	30	20	1.473
2010-11	PRE	9	Normal	-0.453	2.1	0.723	21.7	30	8	0.928
2018-19	PRE	10	Normal	-0.447	5.5	2.117	63.5	30	11	0.827
2019-20	DISR	11	Normal	-0.444	6	2.197	65.9	30	6	0.977
2012-13	PRE	12	Normal	-0.433	5.4	1.467	44	30	1	1.089
2016-17	PRE	13	Normal	-0.428	5.4	2.067	62	30	8	0.845
2013-14	PRE	14	Normal	-0.421	3.9	1.173	35.2	30	1	0.952
2015-16	PRE	15	Normal	-0.401	4.2	1.05	31.5	30	10	1.169

The projected peak rate for 2023–24 under the linear growth model (19.3 per 100,000) reflects a forecasting artifact driven by that season's atypical 52-week reporting duration, which caused the model to extrapolate trend over an extended forecast horizon. This value is retained in the table for completeness but is not interpreted as a meaningful model estimate. The flat model projected peak for 2023–24 (3.23 per 100,000) is not affected by this artifact as it does not extrapolate trend.

Table S6. Isolation Forest contamination parameter sensitivity sweep across three threshold levels, FluSurv-NET, United States, 2009–2025.

Season	Phase	Primary Classification	Robust Anomaly	Primary Score	Contam=0.05	Contam=0.10	Contam=0.15
2017-18	PRE	Anomaly	Yes	-0.623	Anomaly	Anomaly	Anomaly
2024-25	REC	Anomaly	Yes	-0.615	Anomaly	Anomaly	Anomaly
2023-24	REC	Anomaly	Yes	-0.6	Anomaly	Anomaly	Anomaly
2014-15	PRE	Normal	No	-0.569	Normal	Normal	Anomaly
2022-23	REC	Normal	No	-0.553	Normal	Normal	Normal
2021-22	DISR	Normal	No	-0.538	Normal	Normal	Normal
2011-12	PRE	Normal	No	-0.529	Normal	Normal	Normal
2009-10	PRE	Normal	No	-0.516	Normal	Normal	Normal
2010-11	PRE	Normal	No	-0.453	Normal	Normal	Normal
2018-19	PRE	Normal	No	-0.447	Normal	Normal	Normal
2019-20	DISR	Normal	No	-0.444	Normal	Normal	Normal
2012-13	PRE	Normal	No	-0.433	Normal	Normal	Normal
2016-17	PRE	Normal	No	-0.428	Normal	Normal	Normal
2013-14	PRE	Normal	No	-0.421	Normal	Normal	Normal
2015-16	PRE	Normal	No	-0.401	Normal	Normal	Normal
2020-21	DISR	EXCLUDED	N/A — CDC data suppression		EXCLUDED	EXCLUDED	EXCLUDED

All changepoint delta values are near-zero by design. The flat growth specification contains no trend component; candidate changepoints therefore reflect seasonal timing adjustments only and do not indicate discrete rate-level shifts in the pre-pandemic series. The absence of large-magnitude changepoints confirms that no structural breaks in rate level were present in the training data under this specification, which is the expected and correct result for a flat growth model.

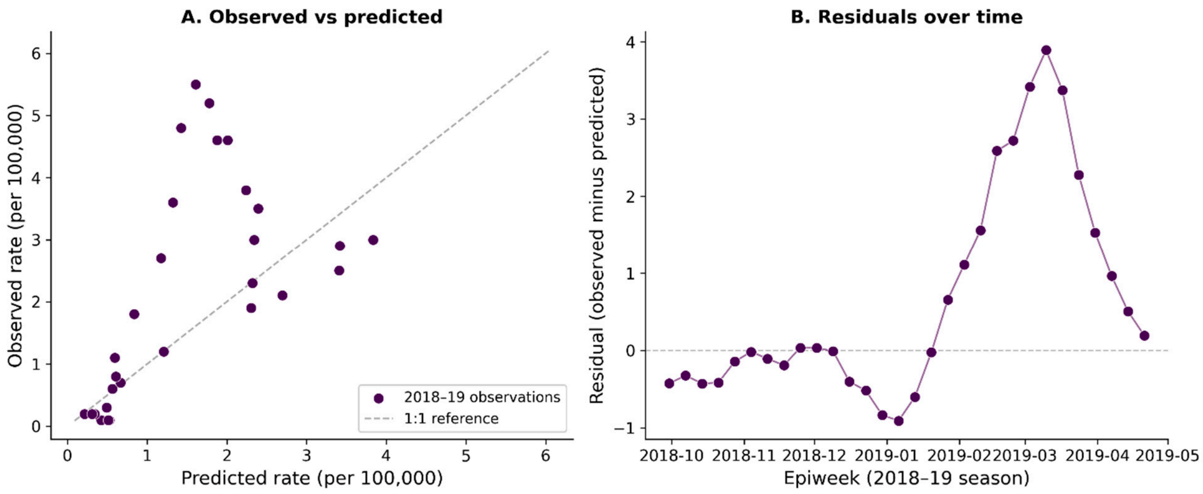

Figure S3. Held-out validation of the primary Prophet flat growth model, 2018–19 season, FluSurv-NET, United States. Panel A: observed versus predicted weekly hospitalization rates (per 100,000 population). The dashed line indicates perfect agreement (1:1 reference). Points above the reference line during peak weeks reflect the above-average severity of the 2018–19 season relative to the training baseline, consistent with conservative underprediction rather than model misspecification. Panel B: residuals (observed minus predicted) over time. The seasonal pattern of residuals, with negative values during the inter-seasonal period and positive values at peak, reflects expected within-season rate variation and shows no evidence of systematic temporal bias. Model trained on 2009–10 to 2017–18 (n = 270 weeks; n = 30 test weeks).

Table S7. Leave-one-out detection date stability, Prophet flat growth model, FluSurv-NET, United States, 2009–2025.

Excluded Season	Training Seasons (n)	First Detection Date	Gap vs Full Model (weeks)	Direction
None (full model)	10	2/2/2020	0	Reference
2009-10	9	2/2/2020	0	Same
2010-11	9	2/2/2020	0	Same
2011-12	9	2/2/2020	0	Same
2012-13	9	2/2/2020	0	Same
2013-14	9	2/2/2020	0	Same
2014-15	9	2/2/2020	0	Same
2015-16	9	2/2/2020	0	Same
2016-17	9	2/2/2020	0	Same
2017-18	9	1/19/2020	-2	Earlier
2018-19	9	2/2/2020	0	Same

Leave-one-out detection date stability analysis, Prophet flat growth model, FluSurv-NET, United States. Each row reflects a model refit with one pre-pandemic season excluded from training (n = 9 seasons per run). First detection date is defined as the first post-training week in which observed rates exceeded the 95% prediction interval upper bound. Gap in weeks is calculated relative to the full-model detection date (2020-02-02). Detection date was identical to the full model in 9 of 10 leave-one-out runs. Excluding 2017–2018, the most anomalous pre-pandemic season, advanced detection by 2 weeks, reflecting a marginally lower training baseline in that run. No leave-one-out specification delayed detection, confirming that the February 2020 finding is not attributable to any single training season.

Table S8. Cross-method convergence of Prophet forecast gaps and Isolation Forest anomaly classifications by season, FluSurv-NET, United States, 2009–2025.

Season	Phase	Prophet Mean Gap	Prophet Gap Direction	IF Rank	IF Anomaly Score	IF Classification	Method Agreement
2009-10	PRE	-0.588	Negative	8	-0.516	Normal	Yes — both methods normal
2010-11	PRE	-0.801	Negative	9	-0.453	Normal	Yes — both methods normal
2011-12	PRE	-1.24	Negative	7	-0.529	Normal	Yes — both methods normal
2012-13	PRE	-0.062	Negative	12	-0.433	Normal	Yes — both methods normal
2013-14	PRE	-0.357	Negative	14	-0.421	Normal	Yes — both methods normal
2014-15	PRE	0.398	Positive	4	-0.569	Normal	Partial — methods diverge
2015-16	PRE	-0.375	Negative	15	-0.401	Normal	Yes — both methods normal
2016-17	PRE	0.541	Positive	13	-0.428	Normal	Partial — methods diverge
2017-18	PRE	1.896	Positive	1	-0.623	Anomaly	Yes — both methods flag
2018-19	PRE	0.588	Positive	10	-0.447	Normal	Partial — methods diverge
2019-20	DISR	0.666	Positive	11	-0.444	Normal	Partial — methods diverge
2021-22	DISR	1.352	Positive	6	-0.538	Normal	Partial — methods diverge
2022-23	REC	0.557	Positive	5	-0.553	Normal	Partial — methods diverge
2023-24	REC	0.541	Positive	3	-0.6	Anomaly	Yes — both methods flag
2024-25	REC	2.68	Positive	2	-0.615	Anomaly	Yes — both methods flag

Cross-method convergence: Prophet forecast gap direction and Isolation Forest classification by season, FluSurv-NET, United States, 2009–2025. Prophet gap direction indicates whether mean observed rates exceeded (Positive) or fell below (Negative) the flat growth model projection. Isolation Forest classification is from the primary model (contamination = 0.10). Method agreement is defined as concordance between a positive Prophet gap and an Isolation Forest anomaly classification, or between a negative gap and a normal classification. Partial divergence reflects the complementary scope of the two methods: Prophet measures deviation from a mean rate baseline while Isolation Forest detects multivariate extremeness across six seasonal features simultaneously. All three robust anomalies (2017–2018, 2023–2024, 2024–2025) show full agreement across both methods. The 2020–21 season was excluded from Isolation Forest scoring due to CDC structural data suppression.
